# An informed approach to the development of primary care pediatric firearm safety messages

**DOI:** 10.1186/s12887-021-03101-4

**Published:** 2022-01-07

**Authors:** Lindsay N. Fuzzell, Sherry Dodd, Sisi Hu, Amanda Hinnant, Sungkyoung Lee, Glen Cameron, Jane M. Garbutt

**Affiliations:** 1 Department of Surgery, Division of Public Health Sciences, Washington University School of Medicine in St. Louis, St. Louis, MO USA; 2 Department of Pediatrics, Washington University School of Medicine in St. Louis, Campus Box 8116, 660 S. Euclid Ave., St. Louis, MO 63110 USA; 3 School of Journalism, University of Missouri, Columbia, MO USA

**Keywords:** Firearm safety, Pediatric primary care, Health communication

## Abstract

**Background:**

Firearm ownership is prevalent in the US and many children spend time in areas where firearms are not stored safely. The AAP recommends firearm safety counseling at pediatric well-visits.

**Methods:**

We developed and tested six contextual messages to promote safe firearm storage based on: absence of harm, collective appeal to understanding child behavior, pediatrician’s authority, evidence-based, fear appeal, and general safety considerations. One hundred four parents who keep firearms at home were recruited from Amazon Mechanical Turk Prime and viewed video messages and reported behavioral intentions and emotional reactions following each message.

**Results:**

All six contextual messages were perceived as important and believable and increased parents’ intentions to follow safety advice provided, but also elicited negative emotions. The authority message elicited more negative emotions and resulted in lower intentions to follow safe storage advice.

**Conclusions:**

Including firearm messages with other child safety advice merits further evaluation. Authority messages should be avoided.

## Background

Firearms are omnipresent in the United States (US) with the equivalent to one firearm for each resident [1, 2]. Studies consistently demonstrate an increased incidence of firearm deaths associated with access to firearms [3]. According to CDC WISQARS (Web-based Injury Statistics Query and Reporting System), in 2019 1,732 children age 17 and under died from a firearm injury (88 (5.1%) unintentional, 946 (54.6%) homicide/legal intervention, 657 (37.9%) suicide, 41 (2.4%) unidentified intent) [4]. If adults stored firearms safely (locked up and unloaded), up to 32% of youth suicides and accidental deaths could be prevented, according to recent research [5].

Pediatricians are a trusted source of health information and are encouraged to provide counseling about firearm safety as a part of well-child care. Specifically, the American Academy of Pediatrics (AAP) recommends that pediatricians routinely screen parents for the presence and availability of firearms in and around the home, and counsel parents who own firearms to prevent access with removal or safe storage (locked up and unloaded) [6]. The AAP advocates that a home without a firearm is the safest home for children and teens, although about 35% of homes with children in the US contain a firearm, and many are *not* locked up and unloaded [7]. This messaging may not be widely accepted, though, as many keep firearms in the home for protection or self-defense and may wish to have the firearm available and loaded at a moment’s notice.

However, few pediatricians counsel parents about firearm safety [8–11]. Providers report barriers to these conversations, including lack of training or time for addressing the topic [12, 13], legal barriers [14], unfamiliarity with firearms, and apprehension about how to broach the subject with parents [15]. Our previous research suggests that pediatricians’ use of the AAP recommended approach to firearm safety is unlikely to be accepted by parents and may offend some, especially firearm owners [10]. Although past work on firearm safety campaigns [16, 17] and video-delivered firearm safety messages [18] is important and useful, little is known about how firearm safety messages could be framed in order to increase parental actions to ensure safe storage, especially among firearm owners or as delivered by providers. Therefore, our objective was to develop firearm safety messages that were acceptable to parents and providers, to be evaluated for effectiveness for safe firearm storage in a future study. This paper reports the third stage in our message development process. Because there is little evidence to predict the impact of different firearm safety messages delivered by healthcare providers, we pose the following research question: How do firearm-owning parents react to different contextual messages about safe firearm storage, and which particular messages are associated with behavioral intentions to adopt providers’ safe firearm storage advice?

## Methods

Our approach to message development involved 3 phases: qualitative interviews with 20 parents and 16 pediatric primary care providers (PCPs) to build message content and identify six themes, an online survey of 23 parents and 23 PCPs to identify acceptable messages, and assessment of 65 firearm-owning parents’ responses to video messages using psychophysiological testing and self-report. (None of the parent or PCP participants were included in more than one stage of the research). Our work was guided by an Advisory Board of key stakeholders (3 providers and 3 parents, including a mix of parents and providers who did and did not own firearms) who were involved in the gathering and interpretation of data, and firearm message development. The first 2 phases are completed and are reported elsewhere [19, 20]. Briefly, contextual messages were developed and winnowed by successive stages of qualitative interviews, advisory board review, survey results, and psychophysiological lab testing. Findings informed refinement of the contextual messages and demonstrated that tailoring messages based on child age was ineffective, and that asking about firearm ownership and providing safety advice consistent with AAP recommendations elicited strong negative cognitive and emotional responses. Informed by these earlier phases of message testing, in the current study we use firearm safety messages that do not ask about firearm ownership, do not recommend removal, and that are not tailored by child age. All study procedures were approved by the University of Missouri Institutional Review Board (IRB), which was utilized as the single IRB for the study.

The goal of the current study was to establish which of the contextual messages were effective in eliciting parents’ intentions to adopt the firearm safety behaviors recommended by the provider. To assess this, each participant viewed six different contextual messages in video format and then responded to items about their behavioral intentions and emotional and cognitive reactions to each of the six messages.

### Participants

Participants were recruited from Amazon Mechanical Turk (MTurk) through TurkPrime between February 25, 2019 and March 5, 2019. MTurk is a web service that enables researchers and businesses to collect data and complete tasks using paid participants or workers. Researchers can set their particular eligibility criteria based on pre-identified participant characteristics. TurkPrime is a research platform designed to improve the quality of the MTurk data collection through preventing multiple participation attempts by the same individual, providing a high level of confidentiality, and other functions for the needs of researchers [21]. Eligible participants were parents of a child under the age of 18, who were located in the U.S., reported attending at least one well-child visit or physical at a pediatrician’s office in the last 3 years, and reported keeping a firearm in or around their home. Informed consent was obtained from all study participants.

### Procedures

Potential participants completed online screening questions to establish eligibility. Each eligible participant viewed six video messages presented in random order via their personal computer. All videos were recorded by the same female physician in the same setting. Each message had the same introduction and concluding safety advice based on general safety statements developed for this study (Table 1), but the content varied to reflect the six contextual messages of interest. These included: 1) *absence of harm* 2) *collective appeal to understanding child behavior* 3) *pediatrician’s authority* 4) *evidence-based* 5) *fear appeal,* and 6) *general safety advice* (Table 1). After the participant viewed each video message, they completed 18 questions to indicate their emotional and cognitive responses. After all 6 messages had been viewed, participants were asked, “What were the videos you just watched generally about?” to assess the validity of their responses. A response which included one or more of the keywords “gun,” “firearm,” or “safety”, was considered a valid response. Each video message was approximately 50 s long and participants spent an average of 20 min completing all study tasks. Participants could self-pace their viewing of videos and completion of survey items. There were no assessment fatigue items. Videos were presented in a random sequence for each participant to avoid response bias in terms of order of video presentation. Participants were compensated $2, a standard rate of pay for mTurk participants [22].

**Table 1:** Message components, and outcome measures with associated items

**Message component**	**Video stated:**
**Introduction**	“There are some safety issues I want to get on your radar, so you can keep your home and places your child visits as safe as possible.”
**Conclusion**	“People have different needs when it comes to firearm storage, and it really isn’t one size fits all. In any home your child spends time in, guns should be inaccessible to curious kids. One of the best ways for firearm owners to do this is to lock up guns and ammunition separate from each other. For folks who keep guns for self-defense and want a loaded gun ready at a moment’s notice, a biometric safe is the next safest option. These safes open with a unique wristband, ring, sticker, or fingerprint.”
**Core contextual message**	**Video stated:**
Absence of harm	“Some people believe that the way they store their guns works because an accident hasn’t happened yet. That is great, but we want to make sure an accident never happens, so let’s review the best practices.”
Collective appeal to understanding child’s behavior	“Whether or not you have firearms, it’s important to make sure your kid is safe in other people’s homes and cars, including friends and family members. It’s OK to say: ‘My kid is curious. I just want to make sure that any firearms are secured.’”
Pediatrician’s authority	“I respect the family traditions of owning guns in America. As your child’s pediatrician, I am your partner in securing a healthy future for your child. This means we need to talk about firearm safety measures wherever your child spends time.”
Evidence-based	“About 19 American kids a day are injured or killed by guns. It’s an alarming number. Let’s make sure your child is protected from being one of those 19 kids with safe firearm storage.”
Fear appeal	“Gun safety is a health issue, even though people don’t often talk about it that way. If being shot does not kill a child, it can lead to a host of other serious health problems. Let’s talk about how to make places your kid spends time healthy and safe in terms of gun storage.”
Safety	“There are many areas of safety that we need to address to keep your child safe and healthy — those include water safety, bike safety, fire safety, and gun safety.”
**Outcome**	**Items**
Message effectiveness	This video was convincing
	This video said something important to me
	Watching this video helped me to feel confident about how to deal with gun safety issues
	Overall, how much did you agree or disagree with what this video said?
	The information in this video is believable to me
Negative emotion	This video made me feel irritated
	This video made me feel angry
	This video made me feel annoyed
Negative cognition	I felt this video was unreasonable
	I felt this video was unfair
	I felt this video was exaggerated
Efficacy/affect	This video was effective at providing advice for keeping kids safe from firearm risks
	This video made me believe that I can keep kids safe from firearm risks
Behavioral intentions	After viewing this video, how likely is it that in the next 3 months, you will:
	Follow firearm safe storage advice provided in this video?
	Talk with friends or family about keeping kids safe from firearms risks?
	Talk to your child’s health care provider about firearm safety?
	Remove firearms from your home, even on a temporary basis?
	Remove firearms from your car (s), even on a temporary basis?

### Measures

For our main outcome measures, we assessed behavioral intentions to complete five firearm safety behaviors: follow firearm safety advice in this video; talk with friends and family about keeping kids safe from firearm risks; talk to child’s healthcare provider about firearm safety; remove firearms from home, even on a temporary basis; and remove firearms from car(s), even on a temporary basis (Table 1). For these measures, respondents used 7-item response scales to indicate their likelihood to adopt the safety behavior (very unlikely, 1, to very likely, 7). An option to report “not applicable because I already store firearms safely according to the advice in the video” was provided.

Outcome measures to assess parents’ emotional and cognitive responses to the contextual messages were adopted from previous studies [23, 24]. Emotional responses included self-reported message effectiveness (5 items about overall quality of the message), negative emotion (3 items), negative cognition (3 items), and response efficacy/affect (2 items about whether the video’s safe storage message would change beliefs). For these measures, respondents used 7-item response scales to indicate their agreement (strongly disagree, 1, to strongly agree, 7) with item statements. Measures were summarized as the mean item responses across scales. All items are displayed in Table 1. We also obtained information about participant characteristics, including parent age, gender, race, income, residential area type, and age of youngest child in the home. Both videos and surveys were shown/administered in English.

### Analytic strategy

To assess the effect of each message on emotional and cognitive responses and intention to adopt recommended firearm safety behaviors, we conducted repeated measures ANCOVAs based on the nature of the within-subject design (all participants viewed the same 6 video messages). Repeated measures ANCOVAs with post-hoc comparisons using LSD (Least Squares Difference) were conducted to compare responses about each contextual message to responses about each of the other contextual messages. Models also included parent gender, residential area type, child age, parent age, and household income. All analyses were conducted using SPSS 26.0.

## Results

Of 120 total completed surveys, 104 participants had valid, consistent, and/or non-duplicated responses that were included in the final sample. Participants ranged in age from 19 to 62 years (median 38 years). The majority were white (82%), female parents/guardians (60%), living in suburban areas (49%), with household incomes exceeding $50,000 annually (65%) (Table 2).

**Table 2:** Participant demographics

		Mean	SD	Range	Percentage	N
Survey time duration (minutes)		18.9	4.96	11.23-35.5		104
Participant age		39.09	10.88	19-62		104
Youngest Child age	0-12 months old				6.70%	7
	1-3 years old				15.40%	16
	3-5 years old				15.40%	16
	5-12 years old				37.50%	39
	12-18 years old				25.00%	26
Gender	Male				40.40%	42
	Female				59.60%	62
Area	Urban				24.00%	25
	Suburban				49.00%	51
	Rural				26.90%	28
Race	White				81.70%	85
	Black or African American				8.70%	9
	American Indian or Alaska Native				1.90%	2
	Asian				3.80%	4
	Native Hawaiian or Pacific Islander				1.00%	1
	Other				1.90%	2
	Prefer not to say				1.00%	1
Income	~ $10.000				0.00%	0
	$10,000-$19,999				5.80%	6
	$20,000-$29,999				9.60%	10
	$30,000-$39,999				8.70%	9
	$40,000-$49,999				10.60%	11
	$50,000-$74,999				26.00%	27
	$75,000-$99,999				25.00%	26
	$100,000 ~				14.40%	15
	Prefer not to say				0.00%	0

The summary of the results of the repeated measures ANCOVAs for all outcome variables is presented in Table 3. The main effects of the contextual messages were statistically significant on: effectiveness, behavioral intention to follow firearm safety advice in the video, to talk with friends or family about firearm risks, and to remove firearms from your home. However, each of the six individual contextual messages did not significantly differ from one another for these outcomes. The main effect of the contextual messages was statistically significant on negative emotion, *F*(4.57, 433.78) = 4.40, *p* = .001, partial η^2^ = .04. The main effect of firearm contextual messages was not statistically significant on behavioral intention to talk to your child’s healthcare provider about firearms, and behavioral intention to remove firearms from your car(s), negative cognition, or perceived efficacy/affect.

**Table 3:** Summary of the results of repeated measures ANCOVAs for all outcomes (*N* = 104)

Predictors	Effectiveness				Negative emotion				Negative cognition				Efficacy				Intention to follow firearm safe storage advice in video				Intention to talk with friends or family about firearm risks				Intention to talk to child’s HCP about firearm safety				Intention to remove firearms from your home				Intention to remove firearms from your car(s)			
	*F*	*df*	*p*	η_p_^2^	*F*	*df*	*p*	η_p_^2^	*F*	*df*	*p*	η_p_^2^	*F*	*df*	*p*	η_p_^2^	*F*	*df*	*p*	η_p_^2^	*F*	*df*	*p*	η_p_^2^	*F*	*df*	*p*	η_p_^2^	*F*	*df*	*p*	η_p_^2^	*F*	*df*	*p*	η_p_^2^
*Predictor*																																				
Firearm Messages (6)	**4.34**	4.55	.001	.04	**4.40**	4.57	.001	.04	1.29	5	.27	.01	1.66	4.57	.15	.02	**2.74**	4.72	.02	.04	**3.29**	4.87	.01	.03	1.13	4.23	.34	.01	**3.09**	4.74	.01	.03	1.69	4.02	.15	.03
*Demographics*																																				
Parent gender (2)	**7.66**	1	.01	.08	3.81	1	.05	.04	**4.25**	1	.04	.04	**7.84**	1	.01	.08	1.70	1	.20	.03	.99	1	.32	.01	2.84	1	.10	.03	**5.93**	1	.02	.06	1.54	1	.22	.03
Residential area (3)	.91	2	.41	.02	1.58	2	.21	.03	.33	2	.72	.01	.87	2	.42	.02	2.96	2	.06	.09	2.58	2	.08	.05	1.65	2	.20	.03	**4.97**	2	.01	.10	**4.97**	2	.01	.15
Child age	1.62	1	.21	.02	**6.92**	1	.01	.07	3.69	1	.06	.04	2.34	1	.13	.02	.46	1	.50	.01	1.44	1	.23	.02	**4.47**	1	.04	.05	**10.69**	1	.001	.10	**9.76**	1	.003	.14
Parent age	.02	1	.89	.00	.02	1	.89	.00	.00	1	.98	.00	.03	1	.86	.00	1.70	1	.20	.03	.01	1	.93	.00	**5.36**	1	.02	.05	**13.02**	1	.000	.12	3.31	1	.08	.05
Household income	**4.42**	1	.04	.04	2.55	1	.11	.03	1.28	1	.26	.01	2.40	1	.12	.03	2.89	1	.09	.05	1.34	1	.25	.01	.01	1	.91	.00	.79	1	.38	.01	1.59	1	.21	.03

*Note.* Degree of freedom(df) for the effect of firearm message are Huynh-Feldt corrected. Statistically significant point estimates are indicated with bold font

The results of post hoc pairwise comparisons for each pair of messages are presented in Tables 4 and 5 for behavioral intentions outcomes and emotion outcomes, respectively. Post-hoc comparison results showed that general safety advice (the *safety* message) (*M* = 5.51, *SE* = .28) elicited significantly greater behavioral intention to follow firearm advice in the video when compared with the *pediatrician’s authority* message (*M* = 5.21, *SE* = .31, *p* = .031). Also, the *pediatrician’s authority* message (*M* = 2.06, *SE* = .17) generated significantly more negative emotions when compared to the *safety* message (*M* = 1.79, *SE* = .14, *p* = .008).

**Table 4:** Pairwise contrasts for adjusted means of behavioral intentions outcomes for each contextual message (*N* = 104)

Outcome	Intention to follow firearm safe storage advice in video		Intention to talk with friends or family about firearm risks		Intention to talk to child’s HCP about firearm safety		Intention to remove firearms from your home		Intention to remove firearms from your car(s)	
Message	***M***_***dif***_ ***(95% CI)***	***p***	***M***_***dif***_ ***(95% CI)***	***p***	***M***_***dif***_ ***(95% CI)***	***p***	***M***_***dif***_ ***(95% CI)***	***p***	***M***_***dif***_ ***(95% CI)***	***p***
*Absence of harm - Collective appeal*	−.12 (−.52, .28)	.55	.01 (−.24, .25)	.96	−.08 (−.38, .23)	.63	−.002 (−.29, .29)	.99	−.25 (−.66, .16)	.23
*Absence of harm - Authority*	.11 (−.27, .50)	.56	.04 (−.20, .27)	.76	−.10 (−.39, .18)	.47	.08 (−.17, .33)	.53	−.10 (−.41, .20)	.50
*Absence of harm - Evidence based*	−.11 (−.53, .30)	.59	.00 (−.21, .21)	.99	−.17 (−.46, .12)	.24	−.14 (−.42, .14)	.31	−.02 (−.29, .25)	.87
*Absence of harm - Fear appeal*	−.09 (−.45, .27)	.61	.20 (−.02, .42)	.08	.08 (−.17, .33)	.53	−.05 (−.31, .21)	.71	−.26 (−.58, .07)	.12
*Absence of harm - Safety*	−.19 (−.60, .21)	.34	.09 (−.12, .31)	.40	−.15 (−.42, .13)	.30	.08 (−.13, .29)	.44	.08 (−.18, .35)	.54
*Collective appeal - Authority*	.23 (−.01, .48)	.06	.03 (−.14, .20)	.73	−.03 (−.27, .21)	.81	.08 (−.19, .35)	.56	.15 (−.08, .38)	.20
*Collective appeal - Evidence based*	.01 (−.25, .27)	.95	−.01 (−.24, .23)	.96	−.10 (−.30, .11)	.35	−.14 (−.41, .13)	.30	.23 (−.20, .66)	.29
*Collective appeal - Fear appeal*	.03 (−.31, .37)	.86	.20 (−.05, .44)	.12	.16 (−.18, .49)	.35	−.05 (−.35, .26)	.76	−.01 (−.35, .34)	.98
*Collective appeal - Safety*	−.07 (−.36, .22)	.62	.09 (−.15, .32)	.47	−.07 (−.33, .19)	.59	.08 (−.14, .31)	.47	.33 (−.04, .71)	.08
*Authority - Evidence based*	−.22 (−.53, .08)	.15	−.04 (−.24, .17)	.73	−.07 (−.26, .13)	.50	−.22 (−.52, .07)	.14	.08 (−.27, .44)	.65
*Authority - Fear appeal*	−.20 (−.52, .12)	.21	.16 (−.06, .39)	.15	.19 (−.10, .47)	.20	−.13 (−.37, .12)	.30	−.15 (−.43, .12)	.27
*Authority - Safety*	**−.31 (−.58, −.03)**	**.03**	.06 (−.17, .29)	.63	−.04 (−.28, .19)	.73	.002 (−.18, .19)	.98	.18 (−.14, .51)	.26
*Evidence based - Fear appeal*	.02 (−.30, .35)	.89	.20 (−.01, .42)	.07	.25 (−.05, .56)	.10	.09 (−.23, .41)	.56	−.24 (−.61, .14)	.21
*Evidence based - Safety*	−.08 (−.35, .19)	.55	.09 (−.08, .26)	.28	.03 (−.17, .22)	.79	.22 (−.04, .48)	.09	.10 (−.19, .40)	.49
*Fear appeal - Safety*	−.10 (−.40, .19)	.49	−.11 (−.31, .10)	.29	−.23 (−.54,.09)	.16	.13 (−.16, .42)	.37	.34 (−.03, .71)	.07

*Note. M*_*dif*_ Difference in adjusted means, *M1-M2* Difference between M1 and M2 (Subtract M2 score from M1 score), *95% CI* 95% Confidence Interval for the Mean Difference

Statistically significant point estimates are indicated with bold font

**Table 5:** Pairwise contrasts for adjusted means of emotion outcomes for each message (*N* = 104)

Outcome	Message Effectiveness		Negative emotion		Negative cognition		Efficacy	
Message	***M***_***dif***_ ***(95% CI)***	***p***	***M***_***dif***_ ***(95% CI)***	***p***	***M***_***dif***_ ***(95% CI)***	***p***	***M***_***dif***_ ***(95% CI)***	***p***
*Absence of harm - Collective appeal*	.02 (−.16, .21)	.79	.01 (−.24, .25)	.97	.03 (−.15, .20)	.75	−.13 (−.33, .06)	.17
*Absence of harm - Authority*	.03 (−.11, .16)	.71	−.08 (−.36, .19)	.56	−.02 (−.20, .15)	.80	−.01 (−.15, .12)	.85
*Absence of harm - Evidence based*	.04 (−.12, .19)	.62	.02 (−.24, .29)	.87	−.09 (−.30, .12)	.37	.05 (−.10, .19)	.55
*Absence of harm - Fear appeal*	.02 (−.11, .16)	.73	.03 (−.27, .32)	.86	−.02 (−.20, .16)	.83	.00 (−.18, .18)	.99
*Absence of harm - Safety*	.02 (−.11, .14)	.82	.19 (−.05, .43)	.12	.13 (−.07, .33)	.21	−.15 (−.32, .02)	.08
*Collective appeal - Authority*	.02 (−.14, .14)	.98	−.09 (−.28, .11)	.37	−.05 (−.21, .11)	.53	.12 (−.03, .27)	.11
*Collective appeal - Evidence based*	.02 (−.16, .19)	.87	.02 (−.15, .19)	.84	−.12 (−.33, .09)	.26	**.18 (.04, .32)**	**.02**
*Collective appeal - Fear appeal*	.00 (−.17, .17))	.99	.02 (−.24, .29)	.87	−.05 (−.24, .14)	.62	.14 (−.07, .34)	.19
*Collective appeal - Safety*	−.01 (−.21, .19)	.93	.18 (−.04, .41)	.11	.10 (−.13, .33)	.39	−.02 (−.23, .19)	.86
*Authority - Evidence based*	.01 (−.15, .18)	.88	.11 (−.09, .30)	.30	−.07 (−.30, .16)	.54	.06 (−.09, .21)	.44
*Authority - Fear appeal*	−.002 (−.12, .12)	.98	.11 (−.17, .38)	.43	.003 (−.18, .18)	.97	.01 (−.19, .21)	.90
*Authority - Safety*	−.01 (−.18, .16)	.90	**.27 (.07, .47)**	**.01**	.15 (−.04, .34)	.13	−.14 (−.34, .06)	.17
*Evidence based - Fear appeal*	−.02 (−.18, .15)	.86	.004 (−.23, .24)	.97	.08 (−.14, .28)	.48	−.05 (−.22, .13)	.60
*Evidence based - Safety*	−.02 (−.19, .15)	.78	.17 (−.03, .36)	.09	.22 (−.01, .46)	.06	**−.20 (−.39, −.002)**	**.048**
*Fear appeal - Safety*	−.01 (−.16, .14)	.90	.16 (−.06, .39)	.12	.15 (−.04, .34)	.13	−.15 (−.35, .18)	.13

*Note. M*_*dif*_ Difference in adjusted means, *M1-M2* Difference between M1 and M2 (Subtract M2 score from M1 score), *95% CI* 95% Confidence Interval for the Mean Difference

Statistically significant point estimates are indicated with bold font

## Discussion

This investigation is the first step towards the development of acceptable and effective messages for pediatric primary care providers to use in discussions with parents to promote firearm safety. It was also one of the first of its kind to explore parents’ attitudes toward video messages about firearm safety. Informed by earlier phases of message testing, we introduced firearm safety advice without asking about firearm ownership or recommending removal. We focused on the possibility of the child’s access to loaded firearms either at home or elsewhere and presented non-judgmental advice about safe storage. This approach is endorsed in the *Connected Kids: Safe, Strong, Secure* program, a program that focuses on reducing unintentional firearm injuries in young children and suicide risk in adolescents [6]. We explored six contextual messages that provided the rationale for action to ensure safe firearm storage. All six messages were perceived as effective and increased these parents’ intention to follow the safety advice provided, although they all elicited negative emotions in these parents (irritation, anger, annoyance). These findings suggest that a pediatricians’ advice about the emotionally charged topic of safe firearm storage might increase safe behaviors.

Our findings provide some insight into approaches to providing firearm safety advice that providers might consider adopting and avoiding. Although the *safety* message was not significantly more effective than the other four arguments, the results did demonstrate that the *pediatrician’s authority* message elicited significantly more negative emotion and was less likely to improve behavioral intentions to follow firearm safe storage advice, as compared to the *safety* message. The *safety* message which includes firearm safety in the context of other common safety concerns (water, bicycle, fire) may resonate more with parents, perhaps because they are accustomed to other safety topics in this setting. The *pediatrician’s authority* message more directly focuses on firearms and tradition using a blunter communication style, which might be off-putting to some parents. Assuming an expert or authority role in this way might offend some patients. These findings concur with prior studies [25], and research that queried firearm owners on trusted messengers to teach about safe storage, with few ranking physicians as good messengers, and instead indicating that law enforcement, hunting/outdoor organizations, and active duty military were the top messengers for this topic [26]. Therefore, we conclude that providers should likely avoid highlighting their authority as the pediatrician and that the *safety* message merits further evaluation to determine if it could influence firearm safety behaviors such as safe storage.

Overall, provider-parent discussion of firearms is challenging at the outset. Parents may view firearm safety as inappropriate in a health context, an intrusion into personal or political matters [19], or may be surprised or annoyed when a provider brings up the topic [27–29]. A recent qualitative investigation reported that firearm owners prefer specific messaging and language surrounding firearms [30]. This includes use of the word “firearm” versus “gun,” a conversation free of political language or undertones, an emphasis on the culture of safety advocated by and for responsible firearm owners, and putting firearm safety in the context of reducing other lethal harms (e.g., medication). Trust in the messenger is also inherently important and many studies have shown that pediatricians are trusted sources of information and advice [28, 30]. Discussing firearm storage is also viewed by providers as a high-stakes topic, as it is inherently value-laden and political in the U.S. [28] Reframing the discussion in light of our findings, with sensitivity around language and values, may make messages more acceptable to providers and parents, leading to more frequent, impactful discussions. Also, non-profit and advocacy organizations (e.g., Sandy Hook Promise; End Family Fire) provide resources and messaging about intentional shootings and/or accidents related to unsafe storage, which may complement clinical communication. Hunting or outdoor organizations, along with law enforcement, may also offer courses that emphasize these same topics [26]. These organizations can also provide information about firearm safes, locks, and biometric solutions, which may provide practical information for at-home use. Biometric solutions, which allow the user to open a safe at a moment’s notice with a fingerprint, unique ring, sticker, or wristband, may be particularly appealing to those who keep a firearm for self-defense.

Although this study is unique in its strengths, it has some limitations. The scope of contextual messages employed in this study is limited, as we used only six, which is not exhaustive in regard to how clinicians may communicate with parents about firearms. Nonetheless, the six contextual messages chosen were based on interviews and a small survey with parents and primary care providers in a previous phase of message development. Further, more than one contextual message can be employed when providers talk about firearm safety, which we did not investigate and warrants further research. Additionally, the messages were recorded/delivered by one White, female pediatrician, which is not representative of the diversity of pediatricians in the U.S. Given the sensitivity of firearm safety messaging, different characteristics of the pediatrician in the video could change the outcome of firearm safety communication, and message delivery by a family’s own pediatrician whom they likely trust and have built a relationship with, could influence outcomes, as well. Participants were overwhelmingly White (~ 82%), as well. U.S. households with children owning firearms are predominately White (43%) [7], though not to this extreme. Finally, although participants reported keeping a firearm in or around their home, they may not have been the owner of the firearm themselves and may have just resided in a home of a firearm owner. Therefore, they may not have been able to act on or control the safe storage of the firearm in the home.

## Conclusions

As trusted sources of health information, primary care providers are a potential source of firearm safety information for parents. There are many barriers to these complex discussions, including being unsure of what to say and being concerned about offending parents. This study aimed to develop and test the acceptability of firearm safety messages with parents who keep firearms in the home. Our findings show that advice from the pediatrician about firearm safety increases parents’ intent to follow that advice. Results suggest that the message is more likely to be followed if it is non-judgmental and contextualized within a *general safety* message (i.e., delivered at the same time as other safety messages such as water, bicycle, and fire safety), while avoiding use of *authority*. This approach needs further testing to establish widespread acceptance and effectiveness in reducing children’s access to loaded firearms.

## Data Availability

The datasets used and/or analyzed during the current study are available from the corresponding author on reasonable request.

## Abbreviations

AAP: American Academy of Pediatrics
ANCOVA: Analysis of covariance
IRB: Institutional Review Board
PCP: Primary care provider
US: United States
η^2^: Eta squared
